# Comparison of three‐dimensional conformal radiotherapy and hepatic resection in hepatocellular carcinoma with portal vein tumor thrombus

**DOI:** 10.1002/cam4.1708

**Published:** 2018-07-31

**Authors:** Fang Su, Kai‐Hua Chen, Zhong‐Guo Liang, Chun‐Hua Wu, Ling Li, Song Qu, Long Chen, Xiao‐Dong Zhu, Jian‐Hong Zhong, Le‐Qun Li, Bang‐De Xiang

**Affiliations:** ^1^ Department of Radiation Oncology Affiliated Tumor Hospital of Guangxi Medical University Cancer Institute of Guangxi Zhuang Autonomous Region Nanning China; ^2^ Hepatobiliary Surgery Department Affiliated Tumor Hospital of Guangxi Medical University Cancer Institute of Guangxi Zhuang Autonomous Region Nanning China

**Keywords:** hepatocellular carcinoma, portal venous tumor thrombus, prognosis, surgical resection, three‐dimensional conformal radiotherapy

## Abstract

**Objective:**

This study aimed to evaluate the safety and efficacy of three‐dimensional conformal radiotherapy (3D‐CRT) and hepatic resection for patients with hepatocellular carcinoma (HCC) involving portal vein tumor thrombus (PVTT).

**Methods:**

We retrospectively analyzed 323 HCC patients involving PVTT. Among them, 134 patients underwent 3D‐CRT, while 189 controls treated with hepatic resection (HR). Survival rate and prognostic analysis were performed using Kaplan‐Meier method and Cox regression analyses.

**Results:**

The 1‐, 2‐, and 3‐year overall survival (OS) of RT group and HR group was 54% vs 62%, 33% vs 47%, and 18% vs 43%, respectively (*P *=* *0.003). In the subgroup of PVTT type analysis, the 1‐, 2‐, and 3‐year OS in RT group was 65%, 39%, and 19%, respectively, while that in HR group was 83%, 53%, and 42%, respectively, in type I PVTT (*P *<* *0.001). The 1‐, 2‐, and 3‐year OS in RT group was 52%, 35%, and 11%, while that in HR group was 55%, 42%, and 25%, respectively, in type II PVTT (*P *=* *0.612). In type III PVTT, the 1‐, 2‐, and 3‐year OS in RT group was 16%, 3%, and 0%, respectively, while that in HR group was 11%, 0%, and 0%, respectively (*P *=* *0.041). Multivariate analysis revealed that tumor size ≥10 cm, Child‐Pugh class B, and type III PVTT are independent predictors of poor prognosis in HCC with PVTT.

**Conclusion:**

3D‐CRT appears to be an effective treatment for patients with HCC involving type II/III PVTT.

## 1 INTRODUCTION

Hepatocellular carcinoma (HCC) is very likely to invade the intrahepatic vessel, especially the portal vein, thus forming the portal venous tumor thrombus (PVTT).^1^ It is reported that the incidence of PVTT is 44%‐62.2%.^2^ PVTT is one of the important biological characteristics and poor prognostic factors of HCC.^3^ Transcatheter arterial chemoembolization (TACE) is one of the most common treatments for advanced HCC patients with PVTT.^4^, ^5^ The median survival time in patients undergoing TACE is remarkably longer than those in conservative treatment group (8.67 months vs 1.4 months); nonetheless, the overall efficacy of TACE remains limited.^6^ The survival time for patients with resectable PVTT is slightly longer.^7^ Cheng et al^8^ had proposed the PVTT classification theory based on different sites in which tumor thrombus invaded the portal vein. They recommended the preferred surgical treatment for patients with type I or II PVTT.^7^, ^8^ However, the efficacy of surgical treatment remains poor for patients with other types of PVTT.^9^

The application of precision radiotherapy technologies, such as 3D‐CRT, IMRT, and SBRT, has rendered radiotherapy a vital role in the comprehensive treatment of HCC combined with PVTT. Rim et al^10^ performed a systematic review to compare the radiotherapy (RT) modalities for hepatocellular carcinoma (HCC) with portal vein thrombosis (PVT), obtained a response rate of 51.3% and 1‐year survival rate of 43.8% for 3D‐CRT. Much stronger evidence for clinical efficacy of radiotherapy has come from a much larger, multicenter study involving 985 HCC patients with PVTT, the PVTT response rate was 51.8%, and median survival time was 10.2 months.^11^ Several additional studies have also suggested that radiotherapy is safe for HCC patients with PVTT and can improve their OS.^12^, ^13^, ^14^ But, which patients are suitable for radiotherapy or surgical treatment remains to be verified in more studies.

This study was thereby conducted to retrospectively analyze the clinical data of HCC patients with PVTT receiving 3D‐CRT or hepatic resection in our hospital. Moreover, the efficacy and safety of radiotherapy in the clinical treatment of HCC accompanying with PVTT were summarized, analyzed, and compared. Besides, the value of radiotherapy and hepatic resection in treating various types of PVTT was further studied in subgroup analysis, so as to provide reference bases for clinical treatment.

## 2 MATERIALS AND METHODS

### 2.1 Ethics statement

This study was approved by the Ethics Committee of the Affiliated Tumor Hospital of Guangxi Medical University [No.CS2017 (32)]. All patients’ information was anonymous.

### 2.2 Patients and patient workup

This study retrospectively reviewed a total of 323 HCC patients with PVTT in Affiliated Tumor Hospital of Guangxi Medical University from January 2000 to December 2014. 134 cases received 3D‐CRT (RT group) and 189 cases treated with hepatic resection (HR group). Patients with HCC had been diagnosed based on diagnostic criteria of the American Association for the Study of Liver Diseases.^15^ The PVTT was diagnosed by characteristic findings of ultrasonography, computed tomography (CT), and/or magnetic resonance imaging (MRI). During the course of surgery or postoperative pathology found tumor thrombus in the portal vein or the main branch were also diagnosed as PVTT. Eligible Patients also had Eastern Cooperative Oncology Group (ECOG) performance status (PS) 0‐1, Child‐Pugh class A or B cirrhosis, and patients received 3DCRT technique. Exclusion criteria were extrahepatic metastases, diffuse intrahepatic lesions, and liver function as Child‐Pugh C.

### 2.3 PVTT types

PVTT could be divided into I‐IV type as described in the previous studies.^8^, ^16^ Classification is shown in Table 1.

**Table 1:** Classification of PVTT

Types
Type I_0_: Tumor thrombi formation found under microscopy
Type I: Tumor thrombi involving segmental branches of portal vein or above
Type II: Tumor thrombi involving right/left portal vein
Type III: Tumor thrombi involving the main portal vein trunk
Type IV: Tumor thrombi involving the superior mesenteric vein

### 2.4 Treatment

The gross tumor volume (GTV) was included in the PVTT and intrahepatic tumors. Planning target volume (PTV) was determined by adding 0.5 cm‐2 cm to GTV. The organs at risk (OARs) were the Normal liver tissue, lungs, kidneys, spinal cord, heart, esophagus, stomach, duodenum, and small bowels. The radiotherapy plan was evaluated by the dose‐volume histogram (DVH). We determine the fraction dose according to the size and location of the tumor. When the target area was small and OARs was well protected, we adopted hypofractionated radiotherapy; fraction dose was 4‐8 Gy, 3 times a week; when the target area was relatively large or irradiation wild adjacent to the OARs, the use of fraction dose was 2‐3 Gy, five times a week. Tumor total irradiation dose was 32‐63 Gy (median 50 Gy), fraction dose was 2‐8 Gy (median 4.0 Gy), the number of irradiation was 6‐25 times (median 11.0 times), 3‐5 times a week. To make the radiation doses comparable, the total dose was converted to biologically effective dose (BED) using an L‐Q model with α/β ratio of 10 Gy; equivalent to conventional radiotherapy dose 50‐91 Gy.

Hepatic resection was performed according to the preoperative assessment of the patients’ various indexes. Specific surgical procedures were described in the previous study.^17^

### 2.5 Follow‐up

The patients were rechecked once every 3 months within the 1st year, once every 6 months within the 2nd to 5th year, and once every year afterward. Each visit included physical examination, complete blood count, serum AFP, blood chemistry, abdominal ultrasound, and CT scan. Recurrence was diagnosed on the basis of two concurring imaging techniques or the combination of increased AFP and consistent ultrasonography or CT findings. Recurrence was defined as a new lesion in the treatment (irradiation/surgery) region or in a liver tissue associated with it; intrahepatic metastasis was a new lesion that occurs in other parts of the liver; a new lesion outside the extrahepatic was defined as distant metastasis.

All metastasis were evaluated for new treatment. Patients with recurrence or metastasis were treated by TACE, radiofrequency ablation therapy, hepatectomy, systemic chemotherapy, or sorafenib therapy. Therapy was decided based on hepatic function, performance status, and economic conditions.

### 2.6 Radiotherapy toxicity and postoperative complications assessment

The Common Terminology Criteria for Adverse Events (CTCAE, v4.0) was applied in observing radiotherapy‐associated acute toxic reactions. Radiotherapy‐associated advanced adverse events were assessed according to the diagnostic criteria of radiation‐induced liver disease (RILD).^18^ The severity of postoperative complications was assessed by reference to the Clavein‐Dindo classification.^19^

### 2.7 Study endpoint

The primary endpoint of the study was survival time after 3D‐CRT and hepatic resection. The survival time was defined as the time between the date of 3D‐CRT or hepatic resection and the date of death. Patients who were alive at the end of follow‐up were censored.

### 2.8 Statistical analysis

The SPSS19.0 software was applied in data analysis and process. Continuous data were expressed as median (range) and analyzed using the Mann‐Whitney *U* test. The significance of differences between categorical data was assessed with the chi‐squared test or Fisher's exact test. Kaplan‐Meier method was adopted to calculate survival rates; log‐rank test was employed for univariate analysis and pairwise comparison between groups. Multivariate analysis was carried out using the Cox proportional hazards model. For all tests, *P* value < 0.05 was considered statistically significant.

## 3 RESULTS

### 3.1 Patients’ characteristics

The general clinical data of the 323 patients are shown in Table 2. As could be seen, the number of patients with the tumor size of ≥10 cm in RT group was remarkably greater than that in HR group. In addition, the numbers of patients with positive HBsAg, cirrhosis, type I, and II PVTT in HR group were greater than those in RT group. Differences in sex, age, alpha fetal protein (AFP), tumor number, Child‐Pugh class, combined with TACE and median survival between two groups were not statistically significant.

**Table 2:** Characteristics of the two groups of patients

Variate	RT group (n = 134)	HR group (n = 189)	*P*
Median age, y (range)	51 (26‐76)	45 (21‐72)	0.170
Gender, M/F	115/19	171/18	0.196
Tumor size			
<10 cm	72	124	0.031
≥10 cm	62	65	
HBsAg, +/−	110/24	172/17	0.018
AFP			
≥400 ng/mL	71	95	0.630
<400 ng/mL	63	94	
Tumor number			
<3	102	134	0.297
≥3	32	55	
Child‐Pugh class			
A	122	181	0.083
B	12	8	
HBsAg			
Positive	91	150	0.020
Negative	43	39	
PVTT type			
I	23	75	<0.001
II	49	77	
III	62	37	
TACE			
Yes	69	115	0.094
No	65	74	
Median overall survival, mo (range)	13 (1‐196)	18 (1‐120)	0.003

AFP: α‐fetoprotein; F: female; HBsAg: hepatitis B surface antigen; M: male; PVTT: portal vein tumor thrombus; TACE: transcatheter arterial chemoembolization.

### 3.2 OS and prognostic factor analysis

**Figure 1:**
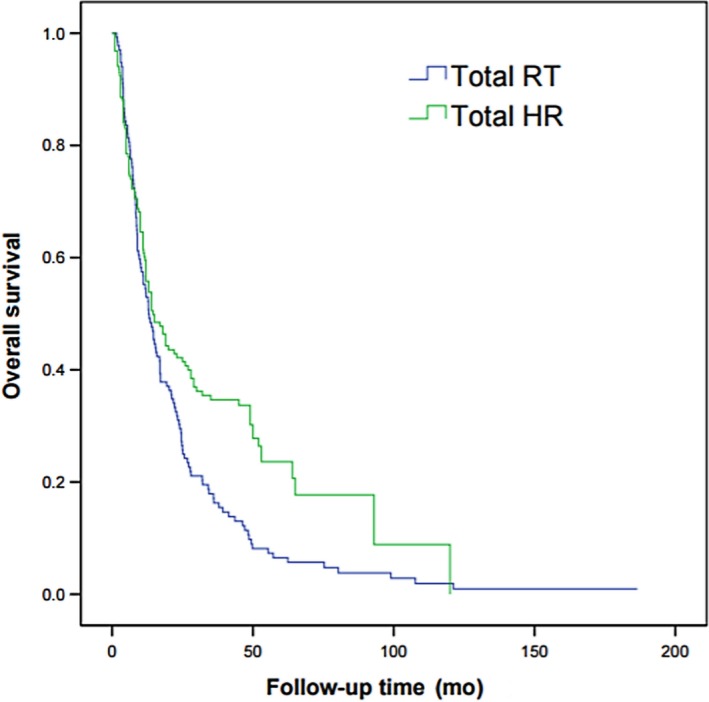
Overall survival curves of patients with HCC involving PVTT treated by 3D‐CRT or hepatic resection (*P* = 0.003)

The median survival of RT group and HR group was 13 and 18 months, respectively. The 1‐, 2‐, and 3‐year OS in RT group was 54%, 33%, and 18%, respectively, which was 62%, 47%, and 43% in HR group. The differences were statistically significant (*P *=* *0.003). The survival curve is shown in Figure 1.

Risk factors related to the prognosis for HCC patients with PVTT include age, sex, tumor size and number, HBsAg, AFP level, Child‐Pugh class, cirrhosis, PVTT type, with/without TACE, and treatment. Results of univariate analysis suggested that, male, tumor size of ≥10 cm, tumor number of ≥3, Child‐Pugh class B, type III PVTT, non‐TACE treatment, and mode of treatment were the factors of poor prognosis for HCC with PVTT (Table 3). Cox multivariate analysis indicated that tumor size of ≥10 cm, Child‐Pugh class B, and type III PVTT were the independent risk factors for survival (Table 4).

**Table 3:** Univariate analysis of prognostic factors

Variate	n	2‐y OS (%)
Age (y)		
≥55	97	25
<55	226	31
Sex		
Male	286	26
Female	37	41
Tumor size (cm)		
<10	196	25
≥10	127	19
Tumor number		
<3	236	28
≥3	87	17
HBsAg		
+	282	26
−	41	29
AFP (ng/mL)		
≥400	166	29
<400	157	32
Child‐Pugh class		
A	303	31
B	20	15
Cirrhosis		
Yes	241	27
No	82	33
PVTT type		
I/II	224	38
III	99	12
TACE		
Yes	184	32
No	139	18
Treatment		
RT	134	25
HR	189	45

AFP: α‐fetoprotein; HBsAg: hepatitis B surface antigen; PVTT: portal vein tumor thrombus; TACE: transcatheter arterial chemoembolization.

**Table 4:** Multivariate analysis of prognostic factors

Variate	Hazard ratio	95% CI	*P*
Male	1.125	0.813‐1.452	0.089
Tumor size ≥ 10 cm	1.409	1.193‐1.827	0.005
Tumor number ≥ 3	1.176	0.901‐1.623	0.274
Child‐Pugh class B	1.502	1.208‐1.798	<0.001
PVTT III	1.638	1.374‐1.913	<0.001
TACE	1.142	0.989‐1.327	0.356
Radiotherapy	1.019	0.875‐1.256	0.135

PVTT: portal vein tumor thrombus; TACE: transcatheter arterial chemoembolization.

### 3.3 Subgroup analysis of PVTT type

**Figure 2:**
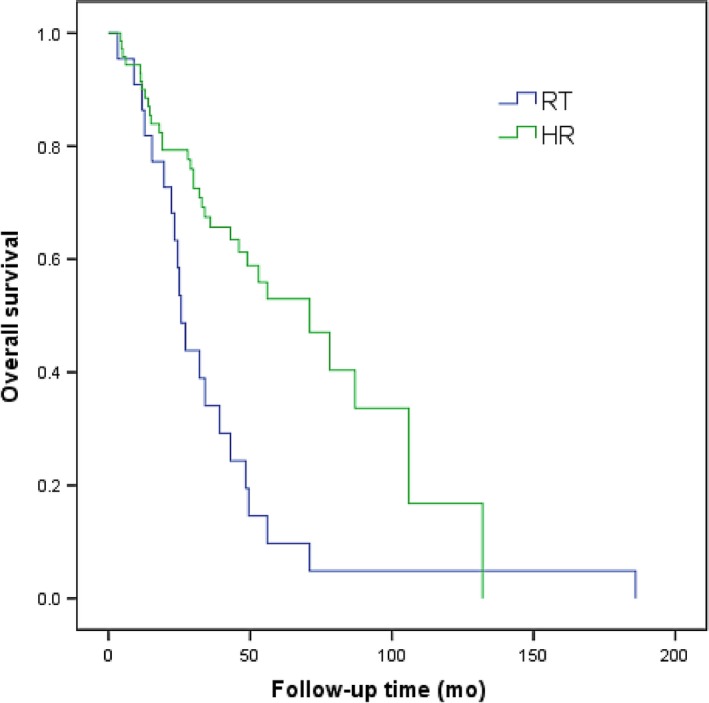
Overall survival curves of patients with HCC involving type I PVTT treated by 3D‐CRT or hepatic resection (*P *< 0.001)

Patients in RT group and HR group were divided into type I, II, and III group according to PVTT type. In patients with type I PVTT, the 1‐, 2‐, and 3‐year OS in RT group was 65%, 39%, and 19%, respectively, while that in HR group was 83%, 53%, and 42%, respectively. The efficacy in RT group was notably lower than that in HR group (*P *<* *0.001; Figure 2).

**Figure 3:**
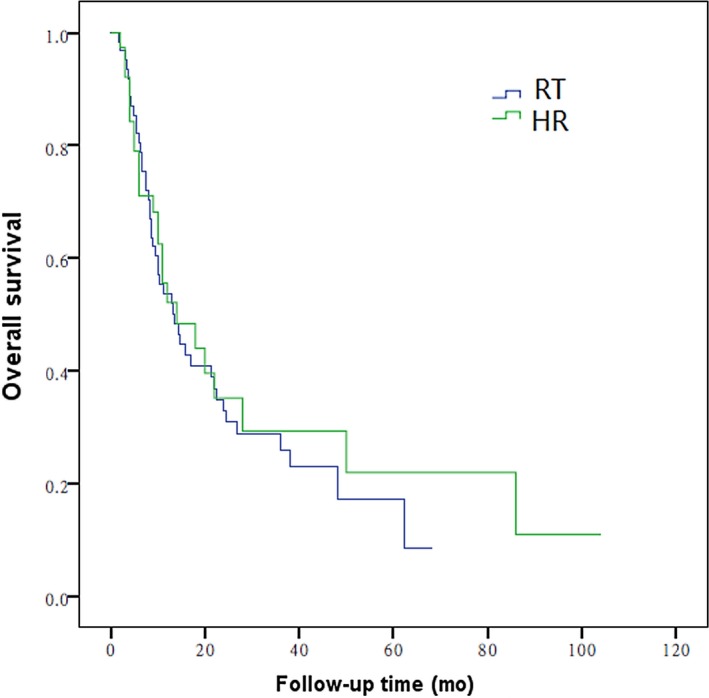
Overall survival curves of patients with HCC involving type II PVTT treated 3D‐CRT or hepatic resection (*P* = 0.612)

**Figure 4:**
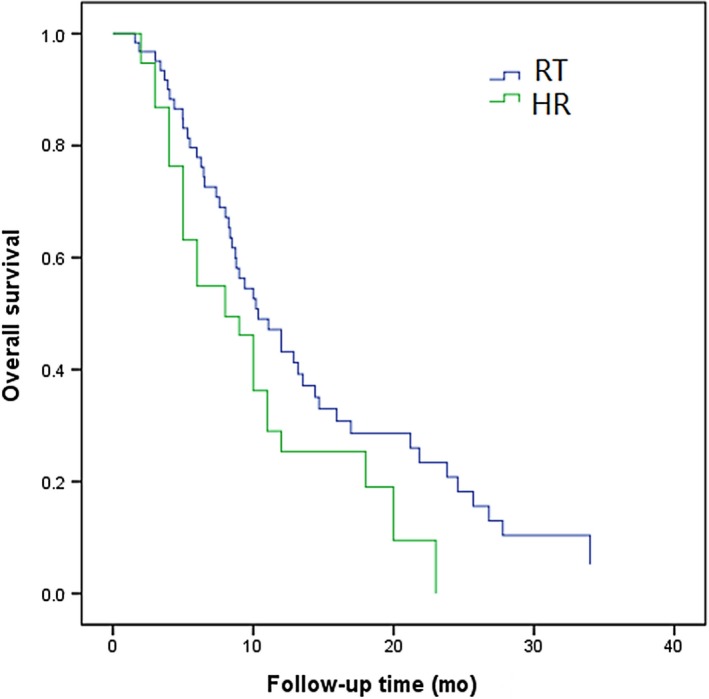
Overall survival curves of patients with HCC involving type III PVTT treated 3D‐CRT or hepatic resection (*P* = 0.041)

In patients with type II PVTT, the 1‐, 2‐, and 3‐year OS in RT group was 52%, 35%, and 11%, respectively, while that in HR group was 55%, 42%, and 25%, respectively. The efficacy between RT group and HR group was not statistically significant (*P *=* *0.612; Figure 3). In patients with type III PVTT, the 1‐, 2‐, and 3‐year OS in RT group was 16%, 3%, and 0%, respectively, while that in HR group was 11%, 0%, and 0%, respectively. The efficacy in RT group was notably higher than that in HR group (*P *=* *0.041; Figure 4). Overall, the prognosis for patients with type III PVTT was poorer than that for those with type I and II PVTT (Table 3).

### 3.4 Subgroup analysis of the combined TACE

**Figure 5:**
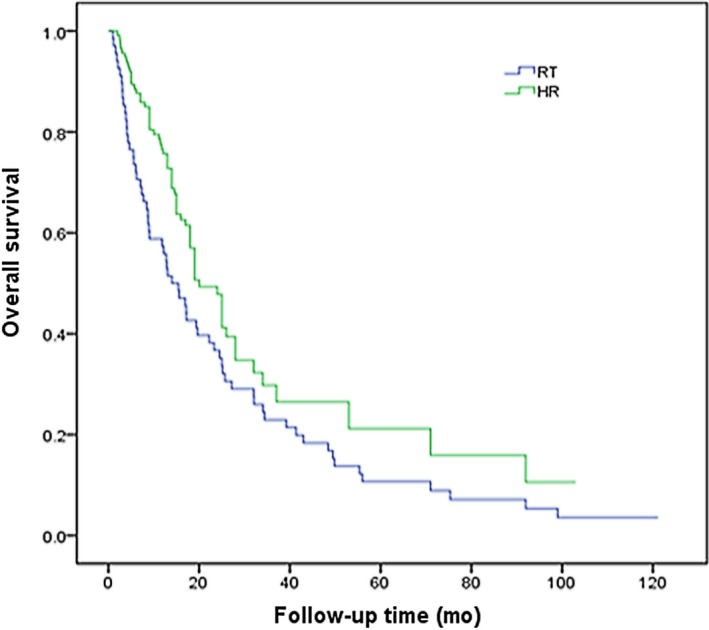
Overall survival curves of patients with HCC involving PVTT treated by 3D‐CRT or hepatic resection combined with TACE (*P* = 0.108)

**Figure 6:**
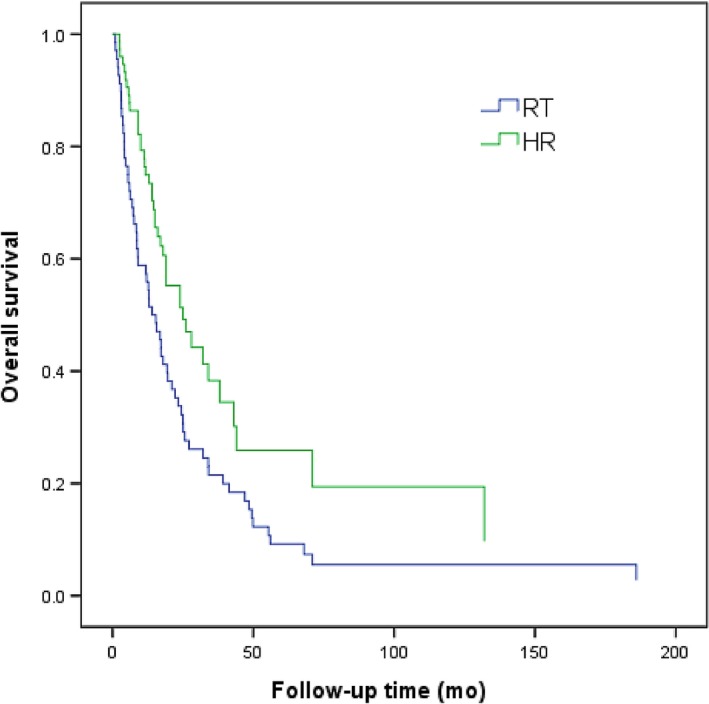
Overall survival curves of patients with HCC involving PVTT treated by 3D‐CRT or hepatic resection without TACE (*P* = 0.018)

Patients in RT group and HR group were divided into combined TACE group and non‐TACE group according to whether TACE had been performed. The results suggested that in HCC with PVTT treated with TACE in combination, the 1‐, 2‐, and 3‐year OS in RT group was 52%, 26%, and 17%, respectively, while that in HR group was 46%, 30%, and 24%, respectively. The efficacy between RT group and HR group was not statistically significant (*P *=* *0.108; Figure 5). In HCC patients with PVTT that did not receive TACE, the 1‐, 2‐, and 3‐year OS in RT group was 31%, 21%, and 9%, respectively, while that in HR group was 45%, 27%, and 18%, respectively. The efficacy in RT group was inferior to that in HR group (*P *=* *0.018; Figure 6). This suggested that TACE was a protective factor for HCC patients with PVTT (Table 3).

### 3.5 Analysis of the cause of failed treatment

During the mean follow‐up of 7.5 (2‐16) years, 114 cases (90%) in RT group had died. The causes of death were as follows, 70 of intrahepatic tumor progression into liver failure, 21 of lung metastases, 3 of peritoneal seeding, 2 of gastrointestinal bleeding, 7 of abdominal lymph node metastases, and 11 of unknown cause.

Additionally, 156 cases (82%) in surgery group had died, including 5 during the perioperative period. Of them, 103 patients died of liver failure caused by intrahepatic tumor progression, five of abdominal lymph node metastases, seven of lung metastases, one of intracranial metastases, and 35 of unknown cause.

### 3.6 Radiotherapy adverse effects and postoperative complications

At the initial period of radiotherapy, some patients developed mild systemic symptoms such as nausea, loss of appetite, and weakness. These symptoms were gradually relieved after radiotherapy adaptation. Most postradiotherapy adverse reactions were grade 1‐2, and 19 cases (14%) were ≥CTCAE grade 3, but these side effects would not cause radiotherapy interruption. In RT group, 7 (5%) patients had radiation‐induced liver disease. These 7 patients had recovered their normal liver function after aggressive liver‐protecting therapy. Two patients had upper gastrointestinal bleeding 3 months and 1 year after the completion of radiotherapy, and they finally died of ischemic shock. Of them, one had esophagogastric varices discovered in gastroscopy, which was considered to be related to cirrhosis.

Severity of postoperative complications in surgery group was evaluated in accordance with the Clavein‐Dindo classification. Most postoperative complications were grade I or II, which were dominated by pulmonary infection (11%) and liver failure (6%).

## 4 DISCUSSION

PVTT is one of the most important prognostic factors of HCC.^20^, ^21^ The average survival time in patients’ naïve to treatment has decreased from 22.4 months to 2.4 months once HCC patients are complicated with PVTT.^22^ The therapeutic strategy for HCC with PVTT remains controversial. A growing number of studies have reported hepatic resection to be safe and effective for selected patients with HCC and PVTT.^7^, ^9^, ^23^, ^24^ The efficacy of surgical treatment in HCC with PVTT is affected by multiple factors, such as the site in which tumor thrombus invades the portal vein, tumor size, intrahepatic tumor dissemination and whether the surgery has achieved radical resection.^25^ Shi et al^7^ had found that OS in type I PVTT patients treated with surgery was notably higher than that in type II and III PVTT patients. Surgical resection is recommended in type I PVTT patients; however, no great progress has been achieved in surgical treatment for patients with type II, III, and IV PVTT.^26^ Despite the studies documenting good postresection outcomes for carefully selected HCC with PVTT, the suitability of the procedure for such patients remains controversial.^27^, ^28^ The extensive application of precision radiotherapy technologies, such as 3D‐CRT,^18^ IMRT,^29^ and SBRT,^30^ has rendered greatly improved role of radiotherapy in the comprehensive treatment of HCC. Plenty of studies have indicated that the median survival time in HCC with PVTT treated by radiotherapy ranges from 8.6 to 44.7 months.^29^, ^30^, ^31^, ^32^, ^33^ Radiotherapy can be employed in unresectable locally advanced HCC patients with no extrahepatic metastasis, those of grade A/B in Child‐Pugh Ratings, and those with the tumor volume being less than 2/3 of normal liver volume.

Some studies suggest that radiotherapy combined with TACE can extend the survival time in HCC patients with PVTT.^31^, ^33^ However, research on whether radiotherapy can benefit various types of PVTT as well as research comparing surgery and radiotherapy is lacking. Tang et al^34^ had retrospectively analyzed the HCC with PVTT treated by 3D‐CRT and surgery, the 1‐year, 2‐year, and 3‐year OS in radiotherapy group were 51.6%, 28.4%, and 19.9%, respectively, which were 40.1%, 17.0%, and 13.6% in surgery group, respectively (*P *=* *0.029). In our research, the 1‐, 2‐, and 3‐year OS in RT group was 54%, 33%, and 18%, respectively, which are markedly lower than those in surgery group (62%, 47%, and 43%, *P *=* *0.003). Results in our research are different from those by Tang et al, which can be mainly attributed to the large proportion of type III tumor thrombus in our study. Subgroup analysis in our study have indicated that the 2‐year OS in type I PVTT receiving 3D‐CRT and surgery are 39% and 53%, respectively (*P < *0.001). This has revealed that surgery is superior to radiotherapy in terms of efficacy. The possible reason is that PVTT has not invaded the vascular wall of portal vein, and surgery can remove the intrahepatic tumor and the PVTT at the same time, which has provided the radical chance for patients. Radiotherapy is similar to surgery in terms of OS for patients with type II PVTT (*P *=* *0.612). The 2‐year OS in patients with type III PVTT after radiotherapy and surgery are 3% and 0%, respectively (*P *=* *0.041), suggesting that radiotherapy is superior to surgical treatment in terms of efficacy. Radiotherapy combined with TACE is recommended for type III PVTT, so as to obtain longer survival time and higher quality of life. However, the prognosis for patients with type III PVTT is poor no matter which treatment is employed, with the median survival time of no more than 6 months. The possible reason is that the type III PVTT will block the portal vein, which has resulted in portal hypertension and hepatic insufficiency. Furthermore, tumor cells are more likely to develop intrahepatic dissemination, which will finally give rise to deterioration as well as hepatic failure and will affect the OS.

In the current study, patients with PVTT receiving TACE can achieve better efficacy than patients who do not undergo TACE, revealing that TACE is a protective factor of HCC with PVTT. HCC with PVTT is frequently combined with multiple lesions in the liver. Small intrahepatic lesions that are less than 1 cm in diameter can hardly be discovered in common CT. Iodized oil deposition on these small lesions has assisted us in discovering these small lesions when TACE is combined. Moreover, it has therapeutic effect. Therefore, it contributes to the implementation of radiotherapy and surgical planning. Considering that 3D‐CRT is similar to surgery in treating type II PVTT, and the radiotherapy has small trauma and mild response, we recommended that radiotherapy is conducted after TACE. For type III PVTT, radiotherapy should be carried out before TACE if the main portal vein has been completely blocked.

Our research also indicated that the toxic reaction of radiotherapy is tolerable for most HCC patients with PVTT. A majority of acute toxic reactions range from degree 1 to 2, which can be alleviated after corresponding symptomatic and supportive treatment. Seven (5%) of the 134 HCC patients undergoing radiotherapy develop radiation‐induced liver disease; nonetheless, the liver functions of patients have recovered to normal levels after active liver‐protecting therapy. Two patients develop upper gastrointestinal hemorrhage 3 months and 8 months after the completion of treatment, respectively. Of them, esophageal‐gastric varices are discovered in 1 case through gastroscopy, which is considered to be related to cirrhosis. These two patients finally die of hemorrhagic shock.

In our research, intrahepatic tumor dissemination and distant metastasis are the major causes of failure in radiotherapy, which is the bottleneck blocking the further improvement of efficacy. Theoretically, radiotherapy combined with sorafenib in treating HCC with PVTT may reduce the probability of intrahepatic tumor dissemination and/or distant metastasis, which can bring greater survival benefits to patients. However, the combination should be used with caution and needs further investigation.^35^

Our study has some limitations. First, this is a retrospective nonrandomized study and thus prone to selection bias. For instance, the majority of RT patients had bigger tumor and type III PVTT, which could lead to worse survival. However, this reflects routine practice where radiotherapy is more commonly offered to patients with bad general function. Second, the frequency of TACE and fractional dose remain sources of controversy, which should be further explored.

From our analysis, RT+ TACE combination treatment or RT monotherapy appears to be an effective treatment for advanced HCC, especially patients with HCC involving type II/III PVTT. To achieve effectiveness of HCC treatment, patients involving PVTT referred for RT+ TACE combination treatment that is associated with better efficacy.

## CONFLICT OF INTERESTS

The authors declare no conflict of interests.
